# Unsupervised [^18^F]Flortaucipir cutoffs for tau positivity and staging in Alzheimer’s disease

**DOI:** 10.1007/s00259-023-06280-7

**Published:** 2023-06-05

**Authors:** Giulia Quattrini, Clarissa Ferrari, Michela Pievani, Andrea Geviti, Federica Ribaldi, Max Scheffler, Giovanni B Frisoni, Valentina Garibotto, Moira Marizzoni, for the Alzheimer’s Disease Neuroimaging Initiative

**Affiliations:** 1 Laboratory of Alzheimer’s Neuroimaging and Epidemiology (LANE), IRCCS Istituto Centro San Giovanni di Dio Fatebenefratelli, 25125 Brescia, Italy; 2 Department of Molecular and Translational Medicine, University of Brescia, 25123 Brescia, Italy; 3 FONDAZIONE POLIAMBULANZA ISTITUTO OSPEDALIERO via Bissolati, 57, 25124, Brescia, Italy; 4 Unit of Statistics, IRCCS Istituto Centro San Giovanni di Dio Fatebenefratelli, 25125 Brescia, Italy; 5 LANVIE – Laboratory of Neuroimaging of Aging, University Hospitals and University of Geneva, 1205 Geneva, Switzerland; 6 Geneva Memory Center, Department of Rehabilitation and Geriatrics, Geneva University Hospitals, 1205 Geneva, Switzerland; 7 Division of Radiology, Geneva University Hospitals, Geneva, Switzerland; 8 Laboratory of Neuroimaging and Innovative Molecular Tracers (NIMTlab), Geneva University Neurocentre, Faculty of Medicine, University of Geneva, 1205 Geneva, Switzerland; 9 Division of Nuclear Medicine and Molecular Imaging, University Hospitals of Geneva, 1205 Geneva, Switzerland; 10 Centre for Biomedical Imaging (CIBM), 1205 Geneva, Switzerland; 11 Biological Psychiatric Unit, IRCCS Istituto Centro San Giovanni di Dio Fatebenefratelli, 25125 Brescia, Italy

**Keywords:** [^18^F]Flortaucipir, Alzheimer’s disease, Cutoff, Gaussian mixture model, Tau PET, Tau positivity

## Abstract

**Purpose:**

Several [^18^F]Flortaucipir cutoffs have been proposed for tau PET positivity (T^+^) in Alzheimer’s disease (AD), but none were data-driven. The aim of this study was to establish and validate unsupervised T^+^ cutoffs by applying Gaussian mixture models (GMM).

**Methods:**

Amyloid negative (A^−^) cognitively normal (CN) and amyloid positive (A^+^) AD-related dementia (ADRD) subjects from ADNI (*n*=269) were included. ADNI (*n*=475) and Geneva Memory Clinic (GMC) cohorts (*n*=98) were used for validation. GMM-based cutoffs were extracted for the temporal meta-ROI, and validated against previously published cutoffs and visual rating.

**Results:**

GMM-based cutoffs classified less subjects as T^+^, mainly in the A^−^ CN (<3.4% vs >28.5%) and A^+^ CN (<14.5% vs >42.9%) groups and showed higher agreement with visual rating (ICC=0.91 vs ICC<0.62) than published cutoffs.

**Conclusion:**

We provided reliable data-driven [^18^F]Flortaucipir cutoffs for in vivo T^+^ detection in AD. These cutoffs might be useful to select participants in clinical and research studies.

**Supplementary information:**

The online version contains supplementary material available at 10.1007/s00259-023-06280-7.

## Introduction

Alzheimer’s disease (AD) is the most common neurodegenerative disorder and is neuropathologically defined by the presence of β-amyloid plaques and tau neurofibrillary tangles (NFTs) [1]. NFTs are closely associated to neurodegeneration and clinical symptoms [2, 3], and they are therefore considered an essential marker for AD diagnosis.

Tau pathology can be in vivo visualized and quantified using positron emission tomography (PET) with ligands affine to NFTs [4, 5]. Among several radioligands developed so far, [^18^F]Flortaucipir showed high sensitivity and specificity to AD-related NFTs [6, 7], and represents the first and, to date, the only tau PET tracer approved by the U.S.A. Food and Drug Administration to support the diagnosis in patients with suspected AD [8, 9]. [^18^F]Flortaucipir uptake correlates with histological findings of AD-related tau pathology [10–12], with phosphorylated tau (p-tau_181_) in the cerebral spinal fluid (CSF) [13, 14], discriminates between preclinical AD and AD-related dementia (ADRD) [11, 12], and is highly predictive of future cognitive decline and conversion of cognitively normal (CN) and mild cognitive impairment (MCI) to AD [15]. Previous studies reported that [^18^F]Flortaucipir regional binding topographically follows the sequential spread of NFTs [16, 17], and highlighted the strong correlation between tracer uptake, cognitive status, and disease progression [15, 17, 18]. Moreover, the presence of NFTs from stage IV onwards is consistent with a neuropathological diagnosis of AD [10, 19, 20]. This indicates that [^18^F]Flortaucipir PET might be extremely useful to identify AD-related pathology, to stage the disease, and to track disease progression in vivo. Along this line, AD clinical trials are starting to include [^18^F]Flortaucipir tau PET positivity (T^+^) status as an eligibility criterion (e.g., the TRAILBLAZER-ALZ trial investigating the donanemab antibody) [21].

Visual assessment of [^18^F]Flortaucipir PET scans is currently the standard method to accurately define T^+^ status and for staging in a clinical setting [10]. However, visual assessment of PET images requires an experienced nuclear medicine specialist, and is thus not suitable for studies that involve large numbers of subjects. This can be particularly limiting in the context of multicenter studies, which generally include hundreds or thousands of scans. Moreover, PET visual assessment is inherently subjective and may be affected by inter-rater variability. These limitations might be mitigated by using automated tools [10]. A recent study [22] reviewed thresholding methods to dichotomize [^18^F]Flortaucipir quantification into normal/abnormal levels, and to discriminate T^+^ and tau negative (T^−^) groups. Even if [^18^F]Flortaucipir quantifications proved to be reliable among studies [23], T status dichotomization did not. Previous approaches resulted in 82 cut-off values across 23 reviewed studies, ranging from 1.13 to 2.79. This variability is likely due to differences in (i) PET images preprocessing steps, (ii) regions-of-interest (ROIs) used to extract the cutoffs (i.e., single ROIs vs composite ROIs that included several regions involved in AD pathology and spread), (iii) clinical and demographic features of the subjects included in the analyzed cohorts, and (iv) different analytic approaches used for cut-off definition (e.g., receiver operating characteristics [ROC] curves vs a defined number of standard deviations above the mean of the reference population) [22]. Importantly, none of the previous works assessed the agreement between T^+^ assignment based on the proposed cutoff and PET visual rating results nor used data-driven approaches (e.g., the Gaussian mixture models). Furthermore, the effect of factors known to impact NFTs load and propagation (e.g., presence of the allele ε4 of the apolipoprotein E gene [APOEε4], increasing age, and female sex) [18, 24–27], has never been tested.

In this study, we applied the GMM [28] on [^18^F]Flortaucipir quantifications to derive unsupervised cutoffs for T^+^ and staging. GMM-based cutoffs were internally and externally validated in two independent cohorts, and compared with published cutoffs [29–31] and visual assessment. Finally, the effect of APOEε4 status, age, and sex on GMM cutoffs was tested [28].

## Material and methods

Data used in the preparation of this article were obtained from the Alzheimer’s Disease Neuroimaging Initiative (ADNI) database and the Memory Center of the Geneva University Hospitals (GMC).

## Participants

### ADNI (internal validation cohort)

The ADNI (https://adni.loni.usc.edu/;https://clinicaltrials.gov/ct2/show/NCT00106899) was launched in 2003 as a public-private partnership, led by Principal Investigator Michael W. Weiner, MD [32]. The primary goal of ADNI has been to test whether serial magnetic resonance imaging (MRI), PET, other biological markers, and clinical and neuropsychological assessment can be combined to measure the progression of MCI and early AD. Each ADNI study site received approval from its institutional reviewed board. Written informed consent was obtained from all research participants. The datasets used from the ADNI database are detailed in the Appendix A of the Supplementary information. Inclusion criteria for the present study were the availability of Mini-Mental State Examination (MMSE) and Clinical Dementia Rating (CDR) scale scores, amyloid and tau PET data, as well as age, sex, and APOE genotype information. Exclusion criteria were the presence of other psychiatric disorder and a non-ADRD diagnosis at the last available visit. Subjects were classified as follows: (i) CN: MMSE≥25, CDR=0, and consistent diagnosis throughout baseline, tau PET, and according to the last available follow-up visits; (ii) MCI: MMSE≥24, CDR=0.5; (iii) ADRD: CDR>0.5. Subjects were then classified as amyloid negative (A^-^)/positive (A^+^) according to established tracer-specific standardized uptake value ratio (SUVr) cutoffs (i.e., [^18^F]Florbetapir: SUVr>1.11 [33]; [^18^F]Florbetaben: SUVr>1.08 [34]; [^18^F]Flutemetamol: SUVr>0.60 [35]).

### GMC cohort (external validation)

To validate the results from ADNI in an independent and clinical cohort, we used data from the Geneva Memory Clinic (GMC) cohort. Participants were enrolled at the Centre de la Mémoire of Geneva University Hospitals (https://www.hug.ch/centre-memoire) from 2014 to 2022 and underwent routine clinical workup, including clinical and neuropsychological testing, and MRI. Additional procedures, such as blood collection, amyloid, tau, and fluorodeoxyglucose (FDG) PET scans, as well as stool and saliva collection, were performed if deemed clinically useful, or in the context of other research projects [36]. MCI and ADRD stages were defined based on respective clinical diagnostic criteria [37, 38]. For the purpose of this study, GMC was considered for the external validation of GMM-based and previously published cutoffs. As for ADNI, inclusion criteria were MMSE≥25, CDR=0 for CN, MMSE≥24, CDR=0.5 for MCI and, CDR>0.5 for ADRD. The definition of A+/A^−^ was done using the same cutoffs as ADNI.

### APOE genotyping

The procedure for APOE genotyping in the ADNI cohort has been previously described [39] (Supplementary information, Appendix A).

### MRI and PET data

#### Acquisition protocols

All MRI scans in both cohorts were performed on 3T scanners. MRI and PET acquisition protocols for both cohorts are reported in Appendix B of the Supplementary information. Amyloid and tau PET data were selected at the same or at the nearest available timepoint.

### Data pre-processing

#### ADNI pre-processing

Pre-processing methods of 3D T1-weighted MRI and [^18^F]Florbetapir, [^18^F]Florbetaben, and ^[^[^18^F]Flortaucipir scan data were reported elsewhere [40]. The amyloid PET SUVr was obtained normalizing the standardized uptake value (SUV) from the mean composite cortical region (frontal, anterior and posterior cingulate, lateral parietal, and lateral temporal cortical regions) to the conventional (non-weighted) whole cerebellum (SupplementaCite ESM.ry information, Appendix A). When amyloid and tau PET scans were performed at different timepoints, we included subjects classified as A^+^ by the closest amyloid PET scan obtained before the tau PET scan, as no conversion to A^−^ would have be expected at any of the future timepoints. Similarly, we included subjects classified as A^−^ by the closest amyloid PET scan obtained after the tau PET scan. To cover the AD continuum, we included A^−^ CN (*n*=235), A^+^ CN (*n*=117), A^+^ MCI (*n*=89), and A^+^ ADRD (*n*=34). Finally, A^−^ MCI (*n*=22) were included as representative of the non-AD pathology.

#### GMC pre-processing

Concerning amyloid PET, the regional amyloid SUVr from the anterior and posterior cingulate, the precuneus, and frontal, temporal, and parietal lobes was automatically quantified by the BRASS, Hermes medical solutions software (https://www.hermesmedical.com/). Participants were classified by applying the cutoffs mentioned above according to the used tracer.

All 3D T1-weighted MRI images were visually inspected and processed using FreeSurfer v7.1 (https://surfer.nmr.mgh.harvard.edu/), while the [^18^F]Flortaucipir standardized uptake values (SUVs) were computed using PETSurfer (https://surfer.nmr.mgh.harvard.edu/fswiki/PetSurfer) (Supplementary information, Appendix C). Finally, A^−^ CN (*n*=29), A^+^ CN (*n*=7), A^+^ MCI (*n*=50), and A^+^ ADRD (*n*=13) for the AD continuum, and A^−^ MCI (*n*=18) for the non-AD pathology were included from the GMC cohort.

#### Regional [^18^F]Flortaucipir SUVr extraction

**Fig. 1:**
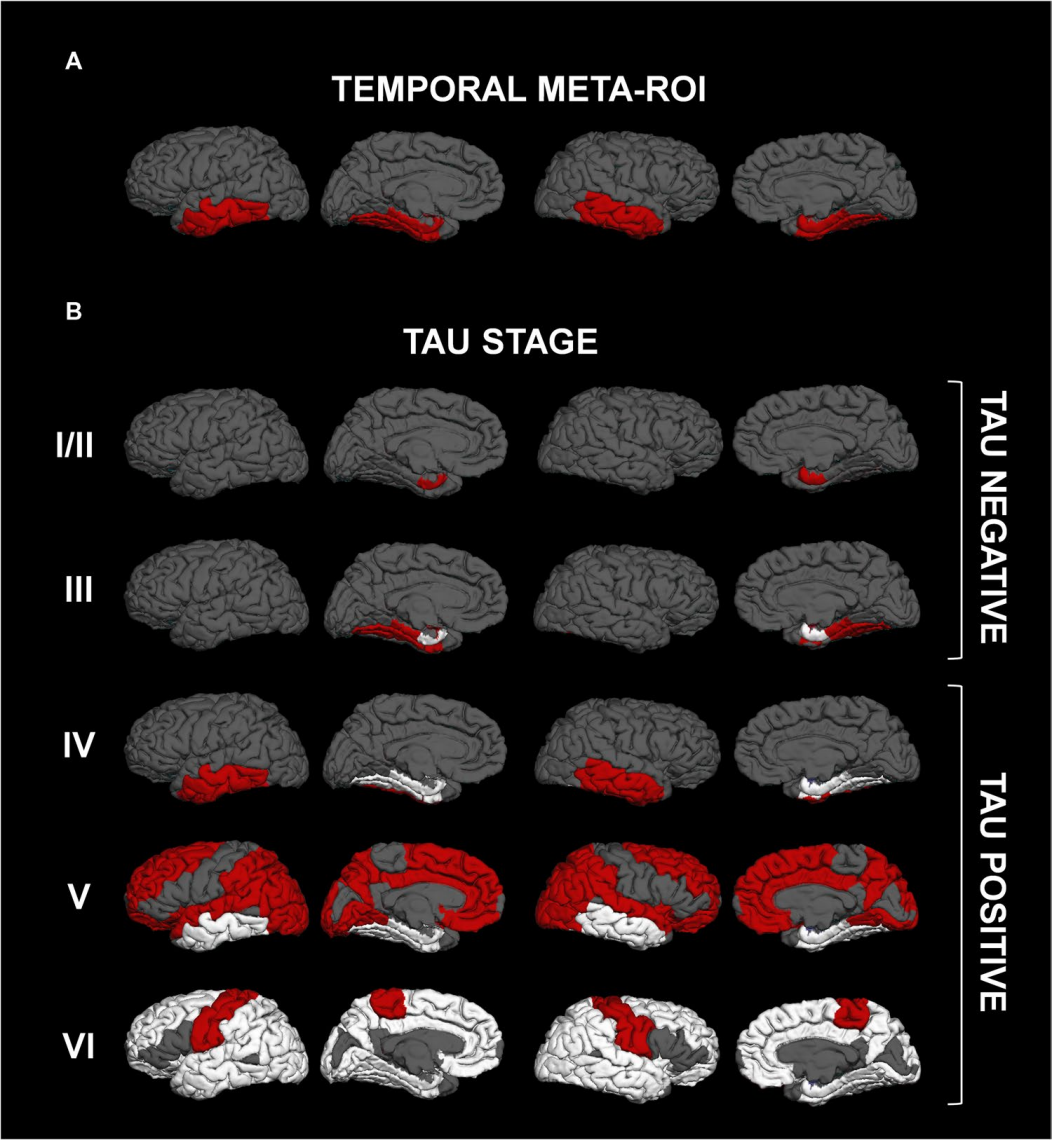
Surface rendering of the temporal meta-ROI (**A**) [30] and of tau spread stages I/II–VI (**B**) [29] used to measure tau-PET uptake. Red regions define areas used for tau-uptake measurement. White regions indicate areas included in the previous tau-spread stage

Regional [^18^F]Flortaucipir SUVr values were derived using FreeSurfer-defined regions-of-interest (ROIs). Specifically, SUVrs were derived in a temporal meta-ROI including those areas most vulnerable to NFTs-related lesions in AD [30, 41] (Fig. 1A), and in the regions corresponding to the tau spread stages (I/II, III, IV, V, and VI) as in Mattsson et al. [29] (Fig. 1B). The hippocampus (stage II) was not included, as [^18^F]Flortaucipir retention signals can be affected in this region by off-target binding of the adjacent choroid plexus [42]. To account for the varying sizes of FreeSurfer-defined ROIs, individual regional SUV weighted means (SUVw) were computed as follows:

$$\textrm{SUVw}=\frac{\left({\textrm{SUV}}_{\textrm{ROI}1}\bullet {\textrm{VOLUME}}_{\textrm{ROI}1}\right)+\left({\textrm{SUV}}_{\textrm{ROI}2}\bullet {\textrm{VOLUME}}_{\textrm{ROI}2}\right)+\left({\textrm{SUV}}_{\textrm{ROI}n}\bullet {\textrm{VOLUME}}_{\textrm{ROI}n}\right)}{\left({\textrm{VOLUME}}_{\textrm{ROI}1}+{\textrm{VOLUME}}_{\textrm{ROI}2}+{\textrm{VOLUME}}_{\textrm{ROI}n}\right)}$$

Then, regional SUVw values were normalized to the conventional (non-weighted) inferior cerebellar gray matter (GM) intensity to obtain regional SUVr values. The highest regional SUVr value from the left and the right hemisphere was selected.

Finally, in the GMC cohort, images were visually rated as positive or negative from a board-certified specialist in nuclear medicine (VG) following published recommendations [10].

### Statistical analyses

Statistical analyses were conducted using the R software package v4.1.1 (R Foundation for statistical computing, https://www.r-project.org/) and the Rstudio GUI (http://www.rstudio.com/; version 1.3.1073). The Kruskal-Wallis test, corrected for multiple comparisons using the Dunn-Bonferroni method, was applied for continuous variable; while the Pearson chi-square test was applied for categorical variables.

For extraction of the cutoffs, the [^18^F]Flortaucipir SUVr of the temporal meta-ROI and individual stages of A^−^ CN and A^+^ ADRD from ADNI were selected. A web-based application (http://www.admodelling.org) [28] was used to apply the GMM on SUVr distributions to detect any underlying subgroups (mixture components), and to define the cutoff (i.e., the value for which the probability of belonging to two consecutive components is equal). To evaluate the possible effect of confounding factors on the derived cut-off, dichotomous (APOEε4: carriers/non-carriers; sex: female/male), or continuous (age: years) covariates were included in each GMM. The integrated completed likelihood criterion was computed to choose the number components of each mixture model, and thus the GMM that best fitted the data [43].

To dichotomize tau status (i.e., T^−^ *vs* T^+^) in ADNI and GMC cohorts, we considered two approaches. In the first one, T^+^ was defined based on GMM and previously published meta-ROI cutoffs [30, 31]. In the second one, each subject was assigned a stage based on GMM and previously published staging cutoffs [29], and T^+^ was defined as stages IV–VI, and T^−^ as stages I/II–III [19, 20] (Supplementary Figure 1), according to the method routinely used in a clinical context. To assess the validity of the cutoffs in identifying tau positivity, in GMC we compared the T^+^ percentages defined by visual assessment (rater 1), GMM-based cutoffs (rater 2) and previously published cutoffs (i.e., rater 3=Jack et al. [30], rater 4=Maass et al. [31], rater 5=Mattsson et al. [29]) using the intraclass correlation coefficient (ICC_2,k_ between rater 1 and each other rater, and values ranging between 0 and 1, with 0 indicating no agreement among raters, and 1 indicating perfect agreement among raters).

Similarly, GMM-based tau staging was compared with that obtained with previous cutoffs and, in GMC, also with visual assessment. The stage assignment was performed as in Maass et al. [31]: first, the T^+^ cutoff for stage VI was applied to identify subjects in stage VI; then, these subjects were removed, and the stage V cutoff was applied to identify subjects in stage V. This approach was continued until stage I/II was reached. Once also the stage I/II subjects were removed, the remaining sample was defined as T^−^ subjects (i.e., not detected NFTs).

## Results

Table 1 summarizes subjects’ features from both cohorts. In the ADNI cohort, participants underwent [^18^F]Florbetapir (64%) or [^18^F]Florbetaben (36%) amyloid PET, while in the GMC cohort participants underwent [^18^F]Florbetapir (46%) or [^18^F]Flutemetamol (54%) scans (Table 1).

**Table 1:** Demographic, clinical, pathological, and genetic features of participants from the Alzheimer’s Disease Neuroimaging Initiative (ADNI) and the Geneva Memory Center (GMC) cohorts

Cohort	A^−^ CN	A^+^ CN	A^−^ MCI	A^+^ MCI	A^+^ ADRD	Test value (*df*,3)	
						*H/X*^*2*^	*p*
ADNI, *n*	235	117	22	89	34		
Age, years	71.9*±*6.7	75.4*±*7.4	79.1±8.2^a^	75.2*±*6.2^a^	78.4*±*9.1^a^	*H*=44.9	**<.001**
Sex (% females)	60%^c,d^	59%^c,d^	32%	43%	56%	*X*^*2*^=13.0	**.011**
Education, years	17.0±2.3^e^	16.8±2.3^e^	17.1±2.1^e^	16.3±2.5	15.2±2.1	H=21.4	**<.001**
MMSE score	29.2*±*1.0^d,e^	29.1*±*1.0^d,e^	28.6±1.7^e^	27.6*±*1.7^e^	21.1*±*3.9	*H*=155.4	**<.001**
CDR score	0.0*±*0.0^d,e^	0.0*±*0.0^d,e^	0.5±0.0^b^	0.5*±*0.0^e^	1.2*±*0.5	*H*=495.9	**<.001**
Amyloid SUVr							
[^18^F]Florbetapir	1.01±0.05	1.29±0.15^a,c^	1.05±0.06	1.43±0.21^a,c^	1.46±0.20^a,c^	*H*=248.9	**<.001**
[^18^F]Florbetaben	1.01±0.04	1.30±0.18^a^	0.99±0.0^a^	1.39±0.21^a^	1.63±0.19^a^	*H*=131.8	**<.001**
%APOEε4 carriers	26%	50%	9%	64%	65%	*X*^*2*^=61.2	**<.001**
GMC, *n*	29	7	18	49	12		
Age, years	70.7±6.9	70.0±8.0	70.2±8.0	74.3±6.7	68.6±8.05	*H*=9.3	.055
Sex (% females)	59%	71%	38%	53%	58%	*X*^*2*^=2.9	.580
Education, years	16.5±4.7^e^	15.3±3.6	14.1±2.9	14.0±3.4	11.3±3.9	*H*=11.5	**.022**
MMSE score	28.9±1.2^c,d,e^	28.3±0.8^e^	26.9±1.6^e^	26.8±1.7	16.8±5.1^e^	*H*=56.1	**<.001**
CDR score	0.0±0.0^c,d,e^	0.0±0.0^c,d,e^	0.5±0.0^e^	0.5±0.0^e^	1.0±0.3	*H*=112.3	**<.001**
Amyloid SUVr							
[^18^F]Florbetapir	1.0±0.1^d,e^	1.3±0.2	1.06±0.03^d^	1.5±0.2	1.5±0.2	*H*=37.9	**<.001**
[^18^F]Flutemetamol	0.5±0.0^b,d,e^	0.9±0.1^d^	0.47±0.05^d,e^	0.8±0.1	0.8±0.1	*H*=46.2	**<.001**

Values are reported as mean *±* standard deviation or percentage (%). *H* chi-square for the Kruskal-Wallis test, *X*^*2*^ Pearson chi-square test, *p* significance level (set to < .050. of the Kruskal-Wallis test. Significance level after the Dunn-Bonferroni correction is reported for comparisons with A^−^ CN (^a^), A^+^ CN (^**b**^), A^−^ MCI (^c^). A^+^ MCI (^d^), A^+^ ADRD (^**e**^), and significant results are reported in bold. *MMSE* Mini-Mental State Examination Test, *CDR* Clinical Dementia Rating Scale, *APOEε4* Apolipoprotein E ε4 allele, *A*^*−*^ amyloid negative, *A*^*+*^ amyloid positive, *CN* cognitively normal, *MCI* mild cognitive impairment, *ADRD* Alzheimer’s disease-related dementia, *n* number, *df* degrees of freedom, *SUVr* standardized uptake value ratio

### Extraction of ^18^F-Flortaucipr SUVr GMM cutoffs

For each region, the best GMM was the one that identified two distinct components (i.e., A^−^ CN-like and A^+^ ADRD-like; Supplementary Table 1) and one SUVr cutoff. The resulting cutoffs were (i) for the temporal meta-ROI, 1.36; (ii) for tau stages, 1.34 (I/II), 1.35 (III), 1.38 (IV), 1.39 (V), and 1.20 (VI).

The adjustment of models for covariates did not improve the goodness of fit index, suggesting that APOE, age, and sex effects on GMMs distribution (and thus on the cut-off estimates) were negligible for cut-off extraction (Supplementary Table 2).

### T^+^ classification

**Fig. 2:**
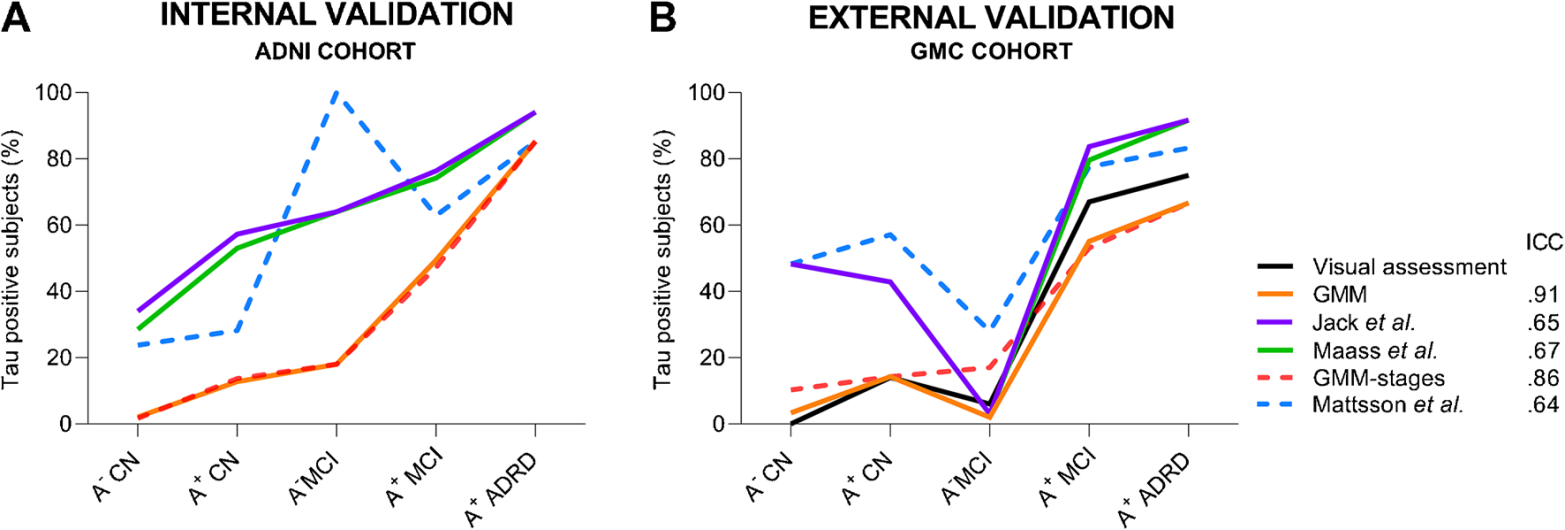
Percentage of subjects classified as tau positive (T^+^) based on different temporal meta-ROI cutoffs, and by grouping stage-based cutoffs (dashed lines). The performance of the GMM and previously published cutoffs was tested in ADNI (internal validation, **A**) and validated in GMC (external validation, **B**). Concordance with the clinical standard (i.e., visual assessment), was tested in the GMC cohort with the intra-class correlation coefficients (ICCs). The Gaussian mixture models (GMM) cutoffs showed the highest agreement with the clinical standard

In ADNI, the percentage of subjects classified as T^+^ using the GMM cutoff for the temporal meta-ROI were 2% and 13% for A^−^ and A^+^ CNs, respectively, 49% for A^+^ MCI, 85% for A^+^ ADRD, and 18% of A^−^ MCI (Fig. 2A, solid orange line). Similar percentages were found for GMC study participants, where subjects were labeled as T^+^ in 3% and 14% of A^−^ and A^+^ CNs respectively, 55% of A^+^ MCI, 67% of A^+^ ADRD, and 11% of A^−^ MCI cases (Fig. 2B, solid orange line). These proportions were confirmed when tau status was calculated based on stages’ dichotomization for both cohorts (Fig. 2, dashed red line). Frequencies of T^+^ according to the previously published thresholds for the temporal meta-ROI (i.e., Jack et al.: SUVr>1.19 [30]; Maass et al.: SUVr>1.20 [31]), and to the dichotomization of stages (i.e., from stage I-II to stage VI: 1.28, 1.23, 1.31, 1.16, 1.09, according to Mattsson et al.) [29], were higher in both cohorts (Fig. 2). Finally, the T^+^ classification using the visual assessment, considered as the clinical standard for T^+^ identification, was 0% and 14% for A^−^ and A^+^ CNs, respectively, 69% for A^+^ MCI, 75% for A^+^ ADRD, and 6% of A^−^ MCI (Fig. 2B). Concordance with the visual assessment was higher for GMM cutoffs (ICC=0.91 for temporal meta-ROI and ICC=0.86 for stages’ dichotomization) than for previous cutoffs (ICC= 0.65 [30], and ICC=0.67 [31] for temporal meta-ROI and ICC=0.64 [29] for stages’ dichotomization) (Fig. 2B).

### Tau staging

**Fig. 3:**
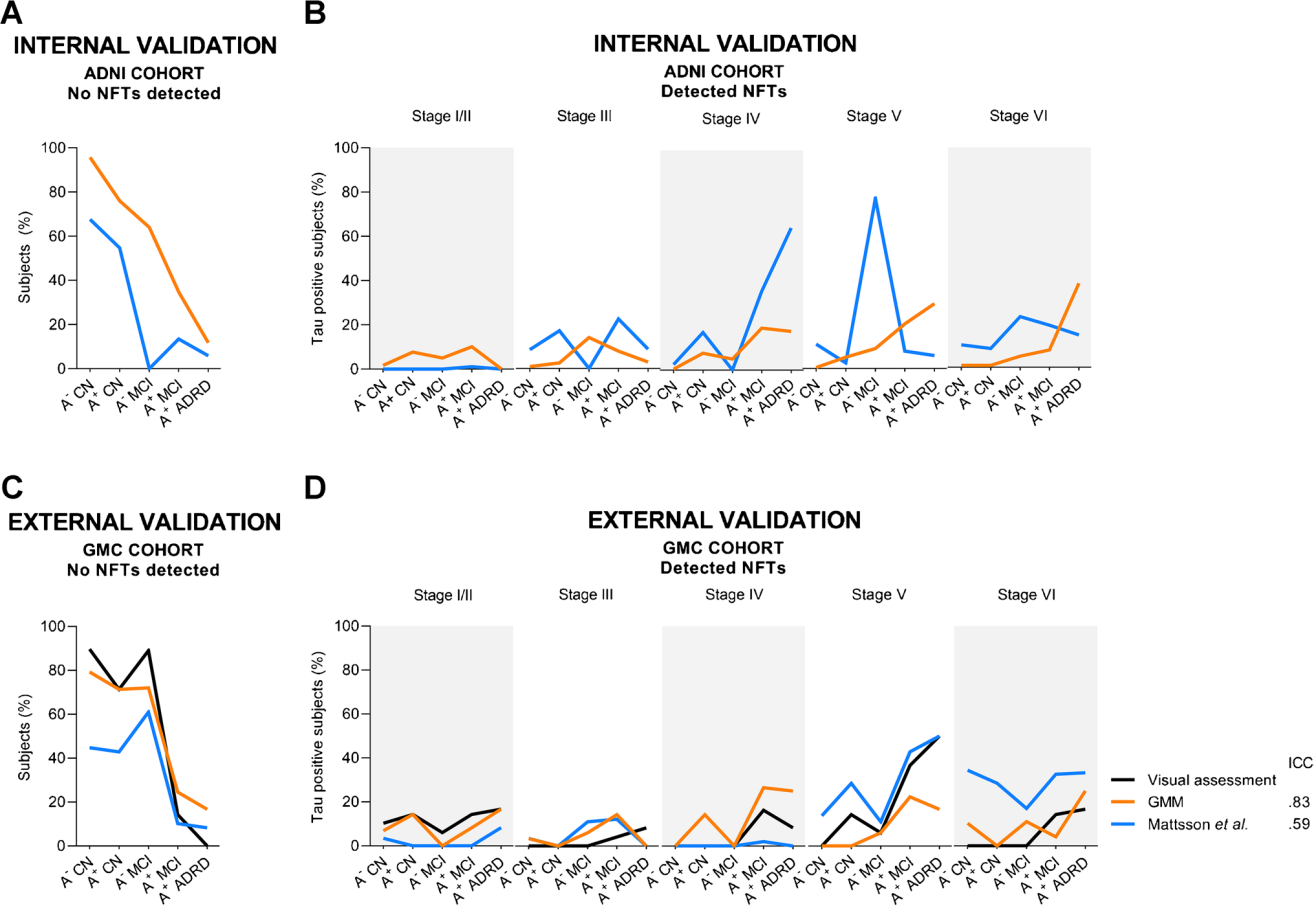
Percentage of subjects classified as tau positive (T^+^) based on the different tau stages’ cutoffs. The performance of the cutoffs was tested in ADNI (internal validation, panels **A**, **B**) and GMC (external validation, panels **C**, **D**). Concordance with the clinical standard (i.e., visual assessment) was tested in the GMC cohort with intra-class correlation coefficients (ICCs; **D**). The Gaussian mixture models (GMM) cutoffs showed the highest agreement with the clinical standard

In ADNI, GMM cutoffs for tau staging showed for subjects classified as free from detectable NFTs a percentage of 96% of A^−^ CN, 76% of A^+^ CNs, 35% of A^+^ MCI, and 64% of A^−^ MCI (Fig. 3A). Among A^+^ MCI, 20% were at stage V, and 19% at stage IV, while A^+^ ADRD were mainly classified as stages VI (38%) and V (29%) (Fig. 3B). According to previously published cutoffs for staging [29], 68% of A^−^ CN, 55% of A^+^ CN, and 13% of A^+^ MCI were classified as free from detectable NFTs (Fig. 3A). The majority of A^+^ MCI patients were classified as stages IV, III, and VI (36%, 23%, 19%), A^+^ADRD patients as stage IV and VI (65%, 15%), and A^−^ MCI as stage V (77%) and VI (23%) (Fig. 3B). The analysis in the GMC cohort confirmed the higher frequency of free from detectable NFTs subjects in the CN and A^+^ MCI groups for GMM cutoffs (A^−^ CN, 79%; A^+^ CN, 71%, A^+^ MCI, 24%), compared to Mattsson et al. cutoffs (A^−^ CN, 45%; A^+^ CN, 43%; A^+^ MCI, 10%) (Fig. 3C), while a lower percentage was reported for A^−^ MCI (i.e., 78% and 61%, for GMM and Mattsson cutoffs, respectively). As in ADNI, Mattsson cutoffs found higher frequencies of T^+^ subjects in stages V (from A^−^ CN to ADRD: 14%, 29%, 43%, 50%) and VI (from A^−^ CN to ADRD: 34%, 29%, 33%, 33%), compared to GMM-based ones (Fig. 3D).

Concordance with the clinical standard (visual assessment) was higher for GMM cutoffs (ICC=0.83) than for Mattsson’s cutoffs [29] (ICC=was 0.59) (Fig. 3D).

## Discussion

The in vivo detection of pathological NFT deposition is of primary importance for AD diagnosis and monitoring [1–3]. Here, we developed data-driven [^18^F]Flortaucipir SUVr cutoffs for the in vivo assignment of tau positivity and tau staging, and validated them in two independent cohorts. Furthermore, we compared the derived cutoffs with previously published thresholds and visual assessment.

Results from ADNI and GMC were consistent with each other and with previous ^[^[^18^F]Flortaucipir PET studies showing that A^−^ CN subjects are usually free of tau pathology, that A^+^ CN might have NFTs deposition beyond the medial temporal region (i.e., from stage IV onwards) [44–47], that half of A^+^ MCI patients were in stage III or lower, and that the majority of A^+^ ADRD were classified from stage III or higher [16, 19, 48]. For A^−^ MCI, frequencies found applying GMM-based cutoffs were similar to those reported by Altomare et al. (11%) in the same cohort [49]. Percentages of T^+^ assigned subjects using GMM-based cutoff for the temporal meta-ROI were also similar to those reported in a previous multi-cohort study applying a similar [^18^F]Flortaucipir threshold (i.e., SUVr=1.34) [46]. Furthermore, the T^+^ and stage assignments according to GMM-based cutoffs were highly consistent with visual assessment and outperformed other cutoffs, revealing an excellent agreement (ICC≥0.85) [50]. The large proportion of A^+^ MCI in stage III or lower (up to 53%) could be explained by the low reliability of [^18^F]Flortaucipir to detect NFTs in the early Braak stages [16, 42, 51], but it might also suggests that these subjects were at a very early stage of the disease or represented the Alzheimer’s and concomitant suspected non-Alzheimer’s pathologic change category [A^+^T^−^ (N)^+^] [1]. To test this possibility, we retrospectively reviewed CSF total tau and p-tau_181_ levels in the ADNI cohort [29, 52, 53]. CSF total tau is a marker of neurodegeneration (N^+^) [1], and p-tau_181_ is sensitive to tau pathology possibly earlier than tau PET [29, 52, 53]. Among the A^+^T^−^ MCI patients classified as stage III or lower using PET, 25 out of 46 had CSF data. Of these, 56% were identified as borderline (20.59–27.41 pg/ml) or T^+^ according to the p-tau_181_ cutoff (27.41 pg/ml) [54] and 100% as N^+^ according to the total-tau CSF cutoff (104.15 pg/ml) [54]. These results confirmed that A^+^/(CSF)T^+^ (PET)T- subjects were likely in an early disease stage, while A^+^/(CSF and PET) T^−^ subjects might represent the A^+^T^-^N^+^ category [1]. GMM cutoffs also identified up to 10% of A^−^ CN subjects (GMC cohort) in stage IV or higher. This might be indicative of false positives or, alternatively, other diseases than AD (e.g., suspected non-AD pathophysiology [SNAP; i.e., A^−^T^+^(N)^−^, A^−^T^−^(N)^+^ or A−T^+^(N)^+^] [55] or primary age-related tauopathy [PART]) [56]. Again, we retrospectively reviewed CSF total tau and p-tau_181_ levels in the ADNI cohort. Data were available for 3 out of 4 A^−^ CN classified as stage IV or higher. For them, the detected patterns were A^−^T^−^(N)^+^ (1 subject) or A−T^+^(N)^+^ (2 subjects), thus confirming that they might represent the SNAP category.

The GMM cutoff for the temporal meta-ROI is consistent with that identified by a recent study [41] assessing the optimal ROI and [^18^F]Flortaucipir SUVr threshold for the differential diagnosis of ADRD *vs* CN and other neurodegenerative conditions. Apart from this recent study, GMM values were generally higher than previously published cutoffs [29–31] and identified a lower number of T^+^ subjects, regardless of diagnostic groups and cohorts, possibly indicating that GMM thresholds are more conservative. This is likely related to the different methods applied for cut-off extraction. To the best of our knowledge, this is the first study that applied a data-driven approach to identify [^18^F]Flortaucipir SUVr values for T^+^ and staging. Previous works used the 95% percentile (i.e., mean plus two standard deviations) based on young [30] or older [29] CN subjects as well as the ROC with Youden index between A^−^ CN and A^+^ MCI and AD [31]. The higher percentages of T^+^ within the A^−^ CN found with the previously published cutoffs [29–31] suggest that the latter group might be more susceptible to false positives than GMM cutoffs. This explanation is supported by the higher agreement of GMM cutoffs with the clinical standard (ICC>0.85), as compared with other cutoffs (ICC<0.62).

In this work, we assessed the effect of the main risk factors for AD (APOEε4 status, increasing age, female sex) on cut-off extraction. Contrary to what was expected, none of these variables affected the cut-off estimations [18, 24, 46]. The lack of effect of risk factors on tau PET positivity cutoffs might be related to a priori selection criteria in the derivation sample. Indeed, while A^−^ CN were mainly younger non-carrier females, A^+^ ADRD dementia cases were generally older carrier males, thus possibly accounting for most of the APOEε4, age, and sex confounding effects. Alternatively, the limited size of the A^+^ ADRD group may have prevented our group from observing any significant effect on cutoffs’ derivation.

The main strength of this work was the application of a probabilistic data-driven approach to derive unbiased cutoffs for [^18^F]Flortaucipir positivity definition. The strength of unsupervised methods lies in their independence from sample features (e.g., diagnostic information), meaning they are not affected by any related potential bias. Secondly, the derived cutoffs were validated in an independent cohort from a memory clinic population, thus supporting generalizability to clinical settings. Finally, the results were validated against visual rating, which is the standard for tau PET assessment in clinical practice. The high agreement between visual rating and GMM cutoffs suggests that these unbiased cutoffs may be a helpful complement to support visual rating in a clinical context. These cutoffs could help physicians in diagnosis by increasing diagnostic confidence [49]. In addition, once anti-amyloid and anti-tau drugs become commercially available, the cutoffs could be used for targeted enrollment of participants according to tau stage. On the other hand, in a research context, they might provide a useful measure of disease stage or progression, which may be useful for both study participant classification and outcome assessment. Finally, as GMM-based cutoffs classified a lower number of T^+^ subjects in the CN group compared to previously published thresholds, they might reduce misdiagnoses and inclusion of non-AD pathologies in future clinical trials. The main limitation of the present study is the small sample size of some subgroups (e.g., A^+^ CN, A^−^ MCI, and A^+^ ADRD), and lacking representation of A^−^ ADRD group. Future studies including larger sample size of these groups, and also considering well-characterized non-AD neurodegenerative diseases, are needed to confirm the GMM cut-off performance. Secondly, the lack of autopsy data prevents the validation of the cutoffs against histologically confirmed amyloid and tau pathology. Indeed, a high but not complete concordance between PET and histology was reported both for amyloid and tau pathology [10, 51, 57, 58], and we cannot exclude that did not have an impact on our results. Also, the possibility of misdiagnoses should be accounted for in both cohorts. Another limiting factor is the lack of follow-up data, which would have offered valuable clinical feedback on the potential usefulness of our cutoffs in terms of disease monitoring and progression tracking. Lastly, despite the fact that [^18^F]Flortaucipir has been approved for staging in AD, we must acknowledge its limited reliability in detecting NFTs in early disease stages.

## Conclusion

The mixture modeling approach enabled the identification of reliable and unbiased [^18^F]Flortaucipir cutoffs for tau positivity and staging supporting their use in both research and clinical settings.

### Supplementary Information

ESM 1 (DOCX 32.7 kb)

## Data Availability

All data and materials support their published claims and comply with field standards. The datasets generated during the current study are available in the ADNI repository (adni.loni.usc.edu/), and on request for the GMC cohort.
