# Supplementary material for: Unsupervised [18F]Flortaucipir cutoffs for tau positivity and staging in Alzheimer’s disease
Source: Eur J Nucl Med Mol Imaging. 2023 Jun 5;50(11):3265–75. doi: 10.1007/s00259-023-06280-7 (PMC10542510; doi:10.1007/s00259-023-06280-7)
Supplement: Supplementary file 1 — (DOCX 32.7 kb) [file 259_2023_6280_MOESM1_ESM.docx]

**Unsupervised [^18^F]Flortaucipir cut-offs for tau positivity and staging in Alzheimer’s disease**

**Supplementary information**

**Appendix A. ADNI datasets**

For this study, we consulted the following datasets from the ADNI repository (https://adni.loni.usc.edu/): APOE genotype, APOERES.csv, [^18^F]Florbetapir SUV, UCBERKELEYAV45_05_12_20.csv; [^18^F]Florbetaben, UCBERKELEYFBB_01_14_21.csv; [^18^F]Flortaucipir, UCBERKELEYAV1451_05_12_20.csv; CSF, UPENNBIOMK10_07_29_19.csv and UPENNBIOMK12_01_04_21.csv.

**Appendix B. MRI and PET protocols**.

*ADNI cohort*

A harmonized acquisition protocol was applied as follows (for details: <http://adni.loni.usc.edu/methods/documents/>): (i) 3D T1-weighted magnetization-prepared rapid acquisition with gradient echo (MPRAGE) images, 1 mm isotropic voxel-size; (ii) amyloid PET, 370 MBq (10.0 mCi)±10%, 20 min (4x5 min frames) acquisition at 50-70 min post-injection ([^18^F]Florbetapir) or 300 MBq (8.1 mCi)±10%, 20 min (4x5min frames) acquisition at 90-110 min post-injection ([^18^F]Florbetaben); (iii) tau PET, 370 MBq (10.0 mCi)±10%, 30 min (6x5min frames) acquisition at 75-105 min post-injection ([^18^F]Flortaucipir).

*GMC cohort*

Acquisition protocols were applied as follows: (i) MRI (SIEMENS MAGNETOM Skyra), 3D T1-weighted MPRAGE images with 1.1 x 1 x 1 mm voxel-size, TR = 1810 ms, TE = 2.19 ms; (ii) amyloid PET, 50 minutes after injection of 200 MBq, 3 5-minute frames ([^18^F]Florbetapir) or 90 minutes after injection of 150 MBq, 4 5-minutes image frames ([^18^F]Flutemetamol); (iii) tau PET, 75 minutes after injection of 180 MBq, 6 5-minutes frames ([^18^F]Flortaucipir).

**Appendix C**. **Pre-processing of imaging data for the Geneva Memory Center cohort.**

All 3D T1-weighted images were visually inspected according to a published rating system [1] and one ADRD and two A^-^ MCI subjects were excluded due to motion artifacts. Images were then processed using FreeSurfer v7.1 (https://surfer.nmr.mgh.harvard.edu/) to compute cortical and subcortical volumes. All outputs were visually inspected, and one A^+^ MCI and one A^-^ MCI cases were excluded due to processing failure. No case required manual editing.

Tau PET data were processed accordingly to the ADNI workflow [2]. [^18^F]Flortaucipir images were first smoothed (8 mm FWMH) using the Statistical Parametric Mapping v12 (SPM 12; <https://www.fil.ion.ucl.ac.uk/spm/>). PETSurfer (<https://surfer.nmr.mgh.harvard.edu/fswiki/PetSurfer>) [3, 4] was then used to extract mean intensity values from ROIs of the Desikan-Killiany atlas. One A^-^ MCI subject was excluded due to processing failure. These values were then normalized to the mean cerebellar grey matter intensity to compute the standardized uptake value (SUV). To reduce the influence of off-target binding, the inferior cerebellar gray matter (GM) was selected as a reference region to compute the [^18^F]Flortaucipir standardized uptake value ratio (SUVr) [5]. Labels from the Spatially Unbiased Infratentorial Template (SUIT) probabilistic normative atlas (50% maximum probability map) [6] corresponding to the inferior cerebellar gray matter (SUIT labels’ indexes: 6, 8-28) were extracted, combined, and binarized using the FMRIB Software Library v6.0 (FSL, <https://fsl.fmrib.ox.ac.uk/fsl/fslwiki/>), and the inclusion mask was created. The individual 3D T1-weighted images were spatially normalized combining the linear (FLIRT, <https://fsl.fmrib.ox.ac.uk/fsl/fslwiki/FLIRT>) [7, 8] and non-linear (FNIRT, <https://fsl.fmrib.ox.ac.uk/fsl/fslwiki/FNIRT>) [9] registrations to the Montreal Neurological Institute (MNI) standard space [10]. Then, for each subject, the reverse-normalization was computed and the inclusion mask was warped onto the individual native space (FNIRT). Tissue-type maps were derived from 3D T1-weighted images using FAST (<https://fsl.fmrib.ox.ac.uk/fsl/fslwiki/FAST>) [11], and the individual GM maps were binarized and overlaid on the respective native inclusion masks, to obtain the inferior cerebellar GM masks. Finally, for each subject, the native [^18^F]Flortaucipir images were affinely aligned and resliced to the respective 3D T1-weighted images, the native inferior cerebellar GM masks overlaid on the [^18^F]Flortaucipir images, and the mean intensity was extracted.

**Supplementary Table 1.** Comparison of Gaussian mixture-models forced to identify 1,2 and 3 components respectively. *G* denotes the number of estimated mixture components. The integrated completed likelihood (ICL) was calculated to select the best model for each stage. Higher ICL denotes a better fit (in **bold**).

| [^18^F]Flortaucipir SUVr regions | G=1  ICL | G=2  ICL | G=3  ICL |
| --- | --- | --- | --- |
| Temporal meta-ROI | 149.1 | **302.5** | 121.1 |
| Stage I-II | 11.3 | **177.1** | 61.6 |
| Stage III | 57.4 | **278.2** | 55.4 |
| Stage IV | 215.8 | **278.8** | 267.4 |
| Stage V | 129.0 | **511.2** | 493.6 |
| Stage VI | 195.6 | **461.9** | 318.5 |

**Supplementary Table 2****.** Mixture model analysis and effects of APOE ε4 allele carriage, age, and sex in the ADNI cohort.

| [^18^F]Flortaucipir SUVr regions | g.o.f index | A^+^ AD component | A^-^ CN component |
| --- | --- | --- | --- |
|  | **BIC^*^** | ***p* value^†^** | ***p* value^†^** |
| *Temporal meta-ROI* |  |  |  |
| No covariates | 321 | NA | NA |
| APOEε4 status (carrier) ^‡^ | 312 | .862 | .201 |
| Age | 328 | .006 | .010 |
| Sex (male) ^‡^ | 311 | .339 | .551 |
| *Stage I/II* |  |  |  |
| No covariates | 209 | NA | NA |
| APOEε4 status (carrier) ^‡^ | 209 | .835 | .001 |
| Age | 207 | .867 | .002 |
| Sex (male) ^‡^ | 202 | .663 | .035 |
| *Stage III* |  |  |  |
| No covariates | 301 | NA | NA |
| APOEε4 status (carrier) ^‡^ | 294 | .633 | .048 |
| Age | 310 | .016 | .031 |
| Sex (male) ^‡^ | 2912 | .277 | .546 |
| *Stage IV* |  |  |  |
| No covariates | 294 | NA | NA |
| APOEε4 status (carrier) ^‡^ | 285 | .715 | .222 |
| Age | 298 | .003 | .003 |
| Sex (male) ^‡^ | 287 | .554 | .053 |
| *Stage V* |  |  |  |
| No covariates | 528 | NA | NA |
| APOEε4 status (carrier) ^‡^ | 521 | .580 | .050 |
| Age | 530 | .813 | <.001 |
| Sex (male) ^‡^ | 527 | .339 | .002 |
| *Stage VI* |  |  |  |
| No covariates | 474 | NA | NA |
| APOEε4 status (carrier) ^‡^ | 464 | .328 | .008 |
| Age | 470 | 400 | .001 |
| Sex (male) ^‡^ | 478 | .765 | .191 |

^*^ Change of BIC less than 15% with respect to BIC of the model without covariates are considered irrelevant for the g.o.f.

^†^ Significance of the covariate effect on each mixture model component of the [^18^F]Flortaucipir SUVr distribution. A covariate has a clear effect on the SUVr distribution if it has a significant effect on both components.

^‡^ In brackets, the category to which the *p* value refers to.

Abbreviations: BIC, Bayesian Information index; g.o.f, goodness of fit; NA, not applicable; SUVr, standardized uptake value ratio; APOEε4, ε4 allele of the apolipoprotein E gene.

**References**

1. Backhausen LL, Herting MM, Buse J, Roessner V, Smolka MN, Vetter NC. Quality control of structural MRI images applied using FreeSurfer—a hands-on workflow to rate motion artifacts. Frontiers in neuroscience 2016;10:558.

2. Jagust WJ, Landau SM, Koeppe RA, Reiman EM, Chen K, Mathis CA, Price JC, Foster NL, Wang AY. The Alzheimer's disease neuroimaging initiative 2 PET core: 2015. Alzheimer's & Dementia 2015;11:757-71.

3. Greve DN, Salat DH, Bowen SL, Izquierdo-Garcia D, Schultz AP, Catana C, Becker JA, Svarer C, Knudsen GM, Sperling RA. Different partial volume correction methods lead to different conclusions: an 18F-FDG-PET study of aging. Neuroimage 2016;132:334-43.

4. Greve DN, Svarer C, Fisher PM, Feng L, Hansen AE, Baare W, Rosen B, Fischl B, Knudsen GM. Cortical surface-based analysis reduces bias and variance in kinetic modeling of brain PET data. Neuroimage 2014;92:225-36.

5. Baker SL, Maass A, Jagust WJ. Considerations and code for partial volume correcting [18F]-AV-1451 tau PET data. Data in brief 2017;15:648-57.

6. Diedrichsen J. A spatially unbiased atlas template of the human cerebellum. Neuroimage 2006;33:127-38.

7. Jenkinson M, Bannister P, Brady M, Smith S. Improved optimization for the robust and accurate linear registration and motion correction of brain images. Neuroimage 2002;17:825-41.

8. Jenkinson M, Smith S. A global optimisation method for robust affine registration of brain images. Med Image Anal 2001;5:143-56.

9. Andersson JL, Jenkinson M, Smith S. Non-linear registration, aka Spatial normalisation FMRIB technical report TR07JA2. FMRIB Analysis Group of the University of Oxford 2007;2:1-21.

10. Andersson JL, Skare S, Ashburner J. How to correct susceptibility distortions in spin-echo echo-planar images: application to diffusion tensor imaging. Neuroimage 2003;20:870-88.

11. Zhang Y, Brady M, Smith S. Segmentation of brain MR images through a hidden Markov random field model and the expectation-maximization algorithm. IEEE Trans Med Imaging 2001;20:45-57.
